# Crystals reveal magma convection and melt transport in dyke-fed eruptions

**DOI:** 10.1038/s41598-020-68421-4

**Published:** 2020-07-15

**Authors:** Helena Albert, Patricia Larrea, Fidel Costa, Elisabeth Widom, Claus Siebe

**Affiliations:** 1 Earth Observatory of Singapore, Nanyang Technological University, Singapore, 639798 Singapore; 2 Central Geophysical Observatory, Spanish Geographic Institute (IGN), 28014 Madrid, Spain; 3 Department of Geology and Andean Geothermal Center of Excellence (CEGA), Facultad de Ciencias Físicas y Matemáticas, Universidad de Chile, Plaza Ercilla 803, Santiago, Chile; 4 Department of Geology & Environmental Earth Science, Miami University, Oxford, OH USA; 5 Dpto. de Vulcanología, Instituto de Geofísica, Universidad Nacional Autónoma de México, Mexico City, Mexico

**Keywords:** Volcanology, Petrology, Geochemistry

## Abstract

The processes and ranges of intensive variables that control magma transport and dyke propagation through the crust are poorly understood. Here we show that textural and compositional data of olivine crystals (Mg/Fe, Ni and P) from the tephra of the first months of Paricutin volcano monogenetic eruption (Mexico, 1943–1952) record fast growth and large temperature and oxygen fugacity gradients. We interpret that these gradients are due to convective magma transport in a propagating dyke to the Earth’s surface in less than a few days. The shortest time we have obtained is 0.1 day, and more than 50% of the calculated timescales are < 2 days for the earliest erupted tephra, which implies magma ascent rates of about 0.1 and 1 m s^−1^. The olivine zoning patterns change with the eruptive stratigraphy, and record a transition towards a more steady magma flow before the transition from explosive to effusive dynamics. Our results can inform numerical and experimental analogue models of dyke propagation, and thus facilitate a better understanding of the seismicity and other precursors of dyke-fed eruptions.

## Introduction

Eruptive sequences of monogenetic eruptions offer a unique opportunity for studying the processes that lead to opening of a volcanic conduit in the upper crust, the birth of a new volcano, and the relation between the eruption deposits and conduit dynamics^1–4^. The configuration of the plumbing system, the amount and composition of volatiles, and the rheological changes due to degassing, cooling, and rapid crystallization all play a role in the eruption dynamics of highly explosive monogenetic eruptions. For example, the Sunset crater eruption (ca. 1085 CE; Arizona, USA) began with weak explosive and effusive activity that was followed by a sub-Plinian explosive phase^1^. In contrast, the Paricutin eruption (1943–1952; Mexico) started with a violent-Strombolian phase that evolved during the following years to a dominantly effusive eruption^5,6^.

The processes of dyke formation and magma transport that may lead to monogenetic eruptions has been investigated by analogue experiments and numerical simulations^7–11^. Analogue experiments investigating dyke propagation show that the stress concentrates at the tip of the dyke during the first stage of the opening, generating instability in the fluid dynamics (complex fluid flow). During this early stage, strain radiates from the growing tip of the dyke with larger horizontal than vertical displacement, and with no incremental strain at the tail, indicating that the dyke opening is sustained^8^. Numerical models of magma transport in dykes have become increasingly sophisticated, with the incorporation of thermal effects of the wall-rock on the physical properties of the magma. These models have focused on the physical requirements to sustain flow in the dyke^10,11^ and characterize the laminar flow in an open conduit^12^. Although these models use equations that are appropriate for a conduit with an already established flow of magma, they are not applicable to the dyke opening process. There is a lack of numerical studies that consider magma convection in a propagating dyke, despite the expectation that magma solidification and melt-back reactions can generate self-mixing and locally influence the magma composition^13^.

Crystals in deposits from monogenetic volcanoes offer additional constraints on the processes that may be involved in the dykes that feed these eruptions. Mineral textural and compositional features, such as rare Mg-Fe oscillatory zoning in olivine^14^, have generally been attributed to open system magmatic processes in the mantle^14^ or at shallower depths^15^. Petrological and geochemical studies of monogenetic eruptions have shown that many magmas contain multiple populations of crystals that imply the existence of shallow and probably ephemeral magma reservoirs^14,16–19^, rather than simply direct transport from the mantle to the surface. Interactions and mixing between multiple magma batches prior to eruption is common, and may be one of the conditions for the magmas to reach the surface and thus the eruption to occur^20^. However, other studies have considered the possibility of convective self-mixing in a magma chamber to explain the variety of mineral compositions and textures^21–25^. For example, a recent study on kimberlitic olivine has attributed multiple concentric growth zones to fast growth and changes in temperature, pressure and oxygen fugacity (*f*O_2_) without needing to invoke magma mixing^26^.

Paricutin volcano is probably the best documented historical monogenetic eruption^27–29^, with a well-preserved stratigraphic record that includes the earliest tephra. This offers the opportunity to study the processes that may be occurring from the beginning of magma transport towards the surface until the end of an eruption. Previous studies have provided petrological and geochemical data for eruptive products spanning the duration of the Paricutin volcano eruption (Mexico, 1943–1952)^5,27,28,30^. Here we focus on understanding the processes recorded in olivine and spinel from the earliest tephra and how they might inform magma transport in the crust, with implications for numerical and analogue experiments of dyke propagation.

## Volcanological and petrological background

The Paricutin eruption began on February 20th, 1943 and was preceded by at least 45 days of seismicity^31^. The eruption started with the ejection of basaltic andesite tephra from a central cone, and evolved compositionally during the following nine years to andesite, mainly erupted as lava from a lateral vent^27^. The different eruptive behaviour between the summit crater (explosive) and the lateral vent (effusive) was probably due to shallow degassing that accompanied the progressive magma differentiation. The onset of the effusive activity at the base of the cone was coincident with the increase of ash production, suggesting an increase of the viscosity and pressurization due to syn-eruptive crystallization^5^. Previous petrological and geophysical studies of the 1943–1944 Paricutin samples have inferred a mean depth of 10 km below the volcano from the magma volatile saturation pressures^27,32^, which matched the hypocentre depth of the precursory earthquakes^31^. Previous studies of the eruption deposits have not reported detailed compositional profiles and textures of the crystals from the earliest erupted tephra, although they have described olivine crystals with different core compositions and normal zoning^32^. The compositions of Cr-rich spinel from Paricutin have been previously reported^29^, and the authors found that they recorded an oxidizing trend with *f*O_2_ between QFM + 0.5 to QFM + 3 (QFM = quartz-fayalite-magnetite reaction as oxygen buffer).

The Paricutin eruption has long been considered a classic example of crustal assimilation combined with fractional crystallization in a calc-alkaline subduction setting^32–36^. However, a recent isotopic study concluded that variations within the 9-year eruption could be explained by melting of a heterogeneous mantle source affected by subduction-related metasomatism, followed by fractional crystallization without significant crustal assimilation^28^.

## Results

### Mineral compositional zoning

The tephra from the first months of the eruption contains (in order of abundance) plagioclase, olivine, spinel, and very rare ortho- and clinopyroxene phenocrysts and microphenocrysts. The phenocryst content varies from about 20 vol% in the earliest tephra to < 10 vol% in the latest, but the sizes of the crystals increase progressively with eruption sequence. The groundmass contains microlites of plagioclase, olivine, and oxides (Figs. S1 and S2). In the early tephra, plagioclase phenocrysts (100–200 µm; measured in the shortest dimension for all the minerals) and microphenocrysts (50–100 µm) comprise ca. 10–15 vol%, olivine phenocrysts (200–300 µm) and microphenocrysts (100 µm) ca. 5–7 vol%, and pyroxene phenocrysts (100–200 µm) < 1 vol%. The latest tephra contains the same minerals except for pyroxene, which is absent, but they are larger and less abundant than in the early tephra. Plagioclase phenocrysts make up ca. 1 vol% and occur mainly as microphenocrysts and in the groundmass. Olivine phenocrysts (300–500 µm) and microphenocrysts (100–200 µm) comprise ca. 2% of the total volume.

**Figure 1:**
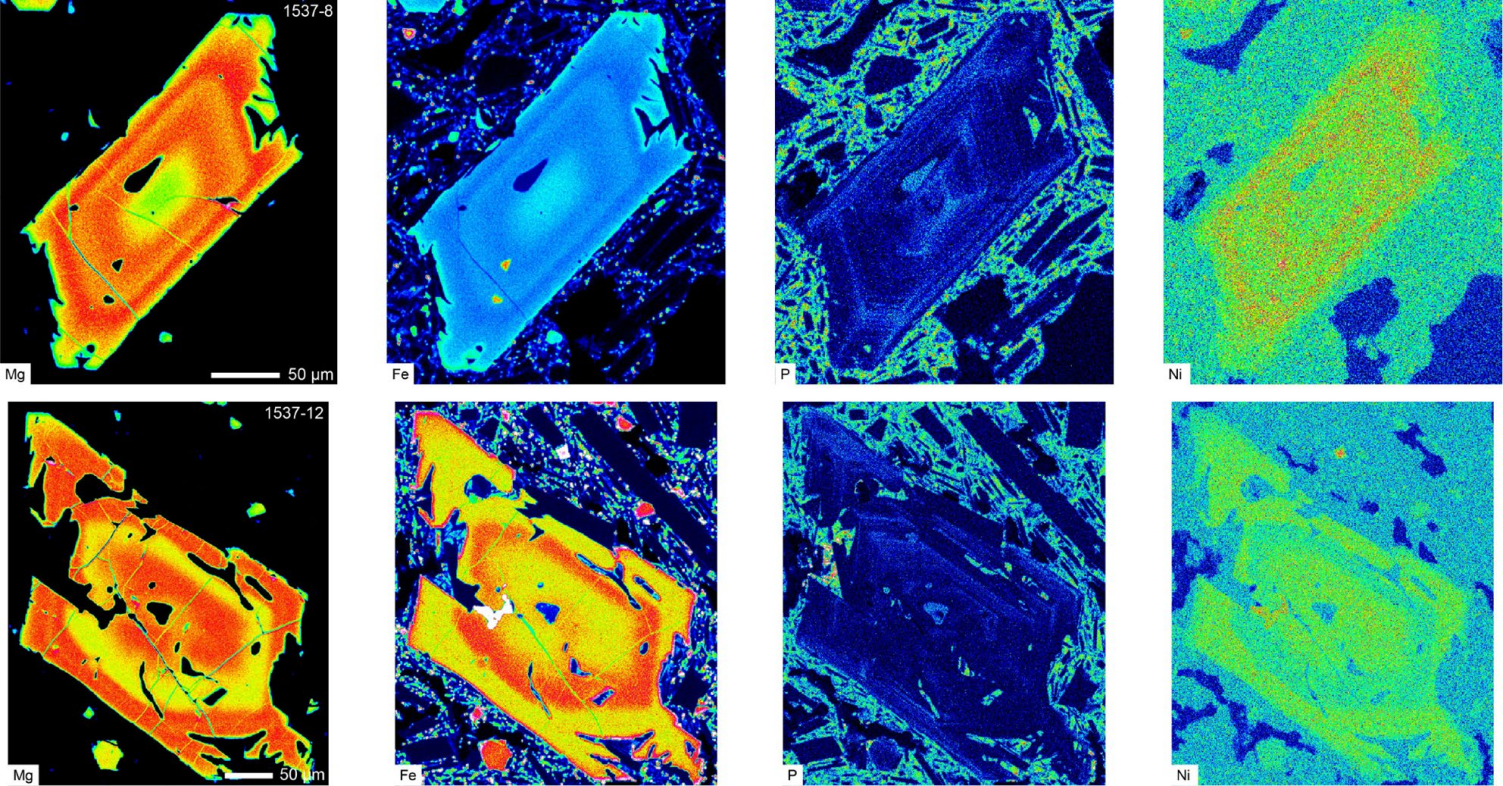
X-Ray compositional maps of magnesium (Mg), iron (Fe), phosphorous (P), and nickel (Ni) of two olivine crystals from the early tephra (sample 1537). The crystals have different core compositions and core to rim patterns, but they both have repetitive and thin zoning of the shown elements. We interpret the zoning to be the result of fast growth within a highly dynamic environment.

**Figure 2:**
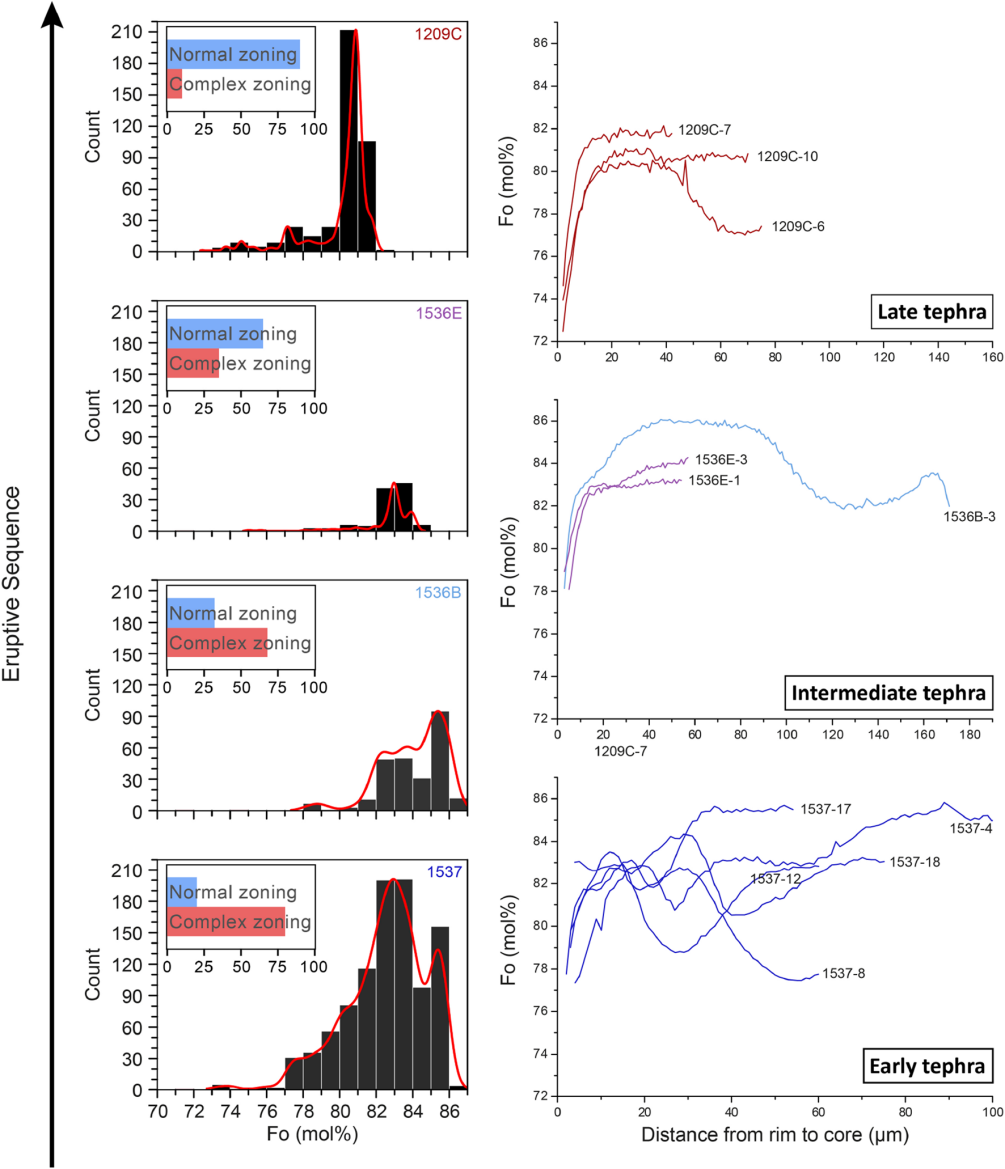
Left panel shows olivine compositional histograms and percentage of complex vs. normally zoned crystals (inset: blue and red bars in %), and right panels are examples of Fo zoning profiles according to tephra samples and eruption sequence. As the eruption progresses, there is an evolution in the olivine composition, proportions of zoning styles, and zoning profile from complex/cyclic to normal.

Olivine occurs in all samples as phenocrysts, microphenocrysts, and microlites (< 50 µm). Most crystals are skeletal and contain spinel inclusions. Olivine compositions range from Fo = 72 to 87 [Fo = 100 × Mg/(Mg + Fe)], with NiO varying from 0.02 to 0.47 wt% and CaO from 0.11 to 0.37 wt%. Compositional traverses across olivine crystals from the early tephra (sample 1537) display a wide variety of zoning patterns in Mg/Fe, Ni, Mn, Ca, P, and Cr. Many crystals (ca. 80 vol%) show oscillating and sharp Fo variations of up to 10 mol% in less than 50 µm (Figs. 1 and S1–S4), and a wide range of core (Fo = 77–87) and rim (Fo = 72–78) compositions. The sharp changes in Fo are also displayed by other elements, including Ni and P (Fig. 1). Moreover, X-ray maps show that the P distribution is indicative of skeletal growth, and that the P and Fo zoning tend to change at the same locations (Fig. S3). The skeletal shape of the olivine, the 2-dimensional distribution of the P zoning, and the roughly similar width of the abrupt compositional changes of P, Fo, and Ni (which have quite different diffusivities^37–39^) suggest that most profiles are the record of fast crystal growth^26,40^ with limited intra-crystal diffusion of Mg/Fe^41^. Thus, these crystals likely grew during magma transport towards the surface. In contrast, most olivine crystals from the latest erupted tephra are normally zoned, with Fo monotonically decreasing from core to rim (82 to 72–78), and only about 10 vol% of complex crystals akin to that of the earliest tephra. The abundance of normally zoned crystals gradually increases (from 20 to 90 vol%; Fig. 2) and that of complex crystals decreases, corresponding to the eruptive sequence. The Fo content sharply decreases within 3–20 μm of the rim of all crystals.

Olivine contains ferroan chromium-bearing spinel inclusions that are zoned, and have Cr_2_O_3_ contents ranging from about 18 to 33 wt%. Spinel composition varies depending on whether they are in the olivine core, mantle, or rim. Some spinel crystals have a concentric zoning pattern that follows their crystal faces and that preserves the external angles (e.g. Figs. S2 and S5). The Mg# [Mg# = 100 × Mg/(Mg + Fe^2+^) mol%] of spinel inclusions shows two crystal groups, one with Mg# = 22–32 and the other with Mg# = 37–45.

Aside from olivine and spinel, the samples contain plagioclase and pyroxene. Plagioclase is the most abundant mineral in the early tephra and phenocryst compositions range from An = 60 to 78 [An = 100 × Ca/(Ca + Na + K) mol%] in the earliest tephra to An = 56 to 74 in the latest tephra. Pyroxenes are rare in all samples. Orthopyroxene crystals are resorbed and some are zoned (Fig. S2) with cores ranging between Mg# = 0.7 and 0.74, and rims of more variable compositions, from Mg# 0.65 to 0.77. Clinopyroxene microphenocrysts are sector zoned and with Mg# = 0.70 to 0.77.

### Diffusion, thermodynamic modelling approaches, and timescales of ascent rates from olivine zoning

**Figure 3:**
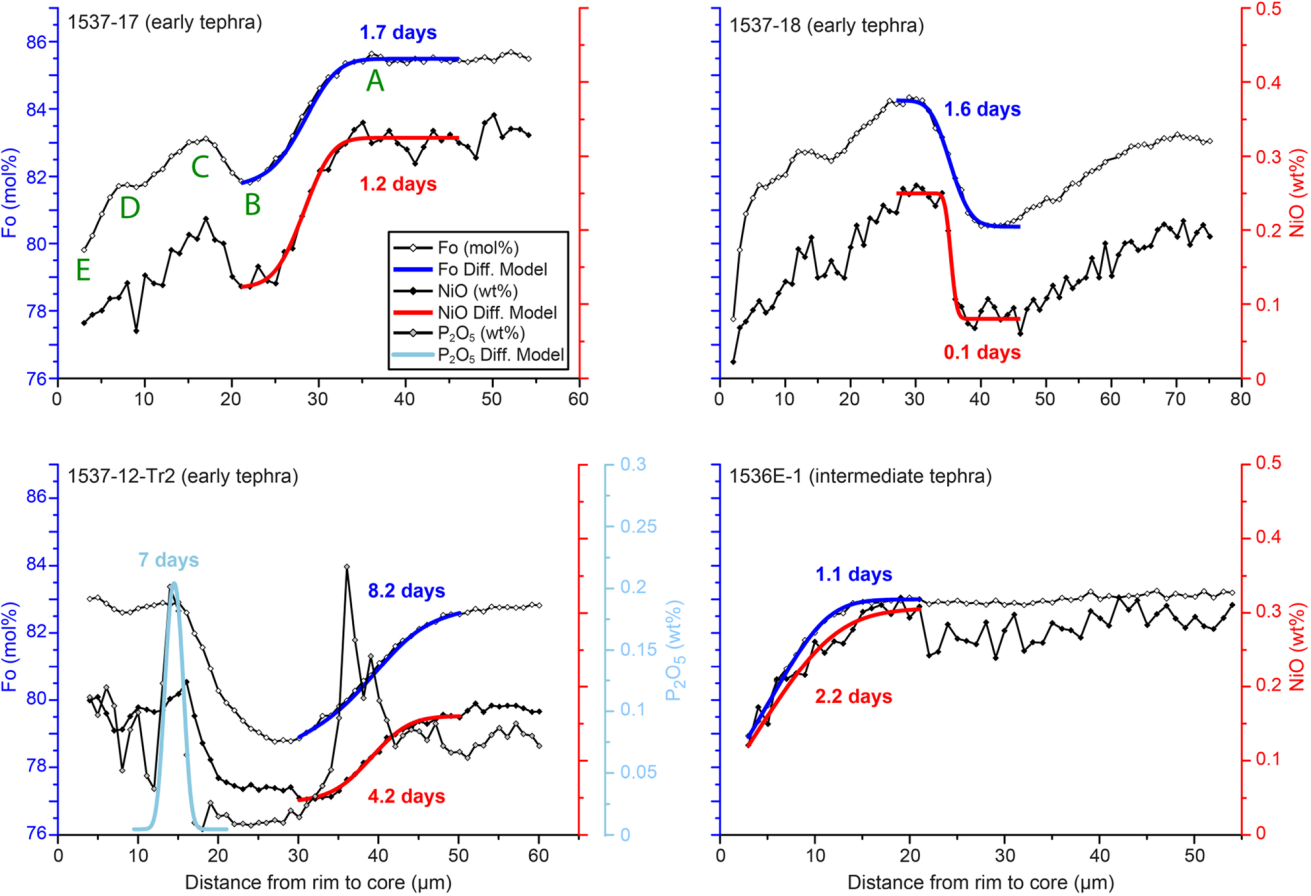
Representative Fo, Ni, and P profiles from the early and intermediate tephra olivine crystals. Locations of the Fo and Ni compositional changes tend to overlap but the Ni profiles are sharper. Calculated timescales are maximum times, since crystal growth contribution is neglected. Letters in green identify different segments of the profile and are used in Fig. 5.

**Figure 4:**
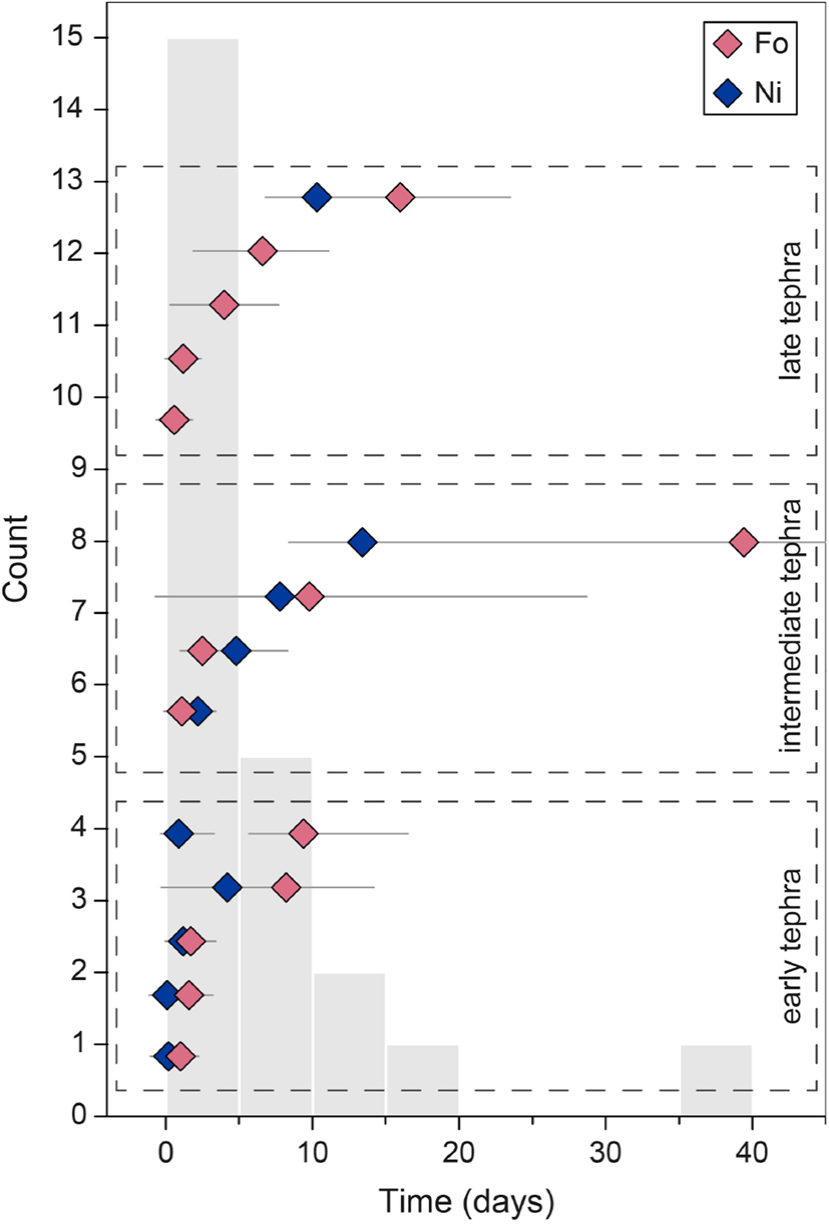
Calculated diffusion times from the early, intermediate, and late tephra plotted as diamonds; each line is a different crystal. In grey, frequency histogram displaying the distribution of Fo and Ni modelling times in 24 olivine crystals. More than 50% of the calculated timescales are less than five days, and less than 1.4 days for the early tephra. Timescales can be found in Table S1.

We modelled the Fo^38^ and Ni^37^ concentration profiles using the software DIPRA^42^ including the effects of diffusion anisotropy (Fig. 4 and Table S1). Olivine crystals display Fo patterns that are broadly a mirror image of the NiO concentrations, but the NiO profiles are sharper than those of Fo. Given that it is difficult to separate in detail the contributions of growth and diffusion in the Fo and Ni profiles, we used NiO as a proxy for the initial profile shape of the Fo, and focused only on portions of the crystals where the geometry of the initial conditions was less ambiguous (Figs. 3 and S6–S9). Abrupt P changes coincide with the inflection point of the Fo and Ni profiles, suggesting that growth was a main contribution to the compositional variations. Thus, the timescales we calculate are maxima, as any growth zoning would increase the length of the profiles. In addition, we have also modelled some of the P zoning profiles^39^ assuming that the width is solely due to diffusion, which in this case is an overestimation of the timescales.

We have attempted to quantify the ranges of temperature and oxygen fugacity recorded by the olivine chemical variations assuming equilibrium crystallization and a range of fractional crystallization models using the Rhyolite-MELTS v.1.2.0. algorithm^43,44^. For this we have used the bulk-rock composition of the sample that is believed to be the closest to the primary magma (sample 1537: 7.47 wt% MgO and 180 ppm Ni)^28^. Isobaric fractional crystallization models have been calculated at different pressures (0.5–5 kbar), with temperature decrease in steps of 5 °C, variable water content (1–4.5 wt%), and *f*O_2_ buffers ranging from QFM + 3 (3 log units above the quartz-fayalite-magnetite buffer) to QFM – 5 (Table S2). We tracked how the Fo content of the crystallizing olivine changed with temperature, *f*O_2_, pressure, and water content, and found that temperature and *f*O_2_ have the strongest influence on Fo content (Fig. S10). Considering the pressure, *f*O_2_ and water variations, the range of olivine Fo and plagioclase An values can be reproduced within a temperature range of 1,120 ± 30 °C. Such temperature is consistent with previous experimental results (T = 1,000 to 1,150 °C^45^) and field temperature measurements (1,110 °C in December 1944^46^). The estimated temperature error encompasses the uncertainty that might be derived from variations in the *f*O_2_ on the diffusion modelling.

Modelling the Fo and Ni concentrations gives timescales of approximately less than one to ten days for all crystals from the early tephra, one day to about a month for crystals from the intermediate tephra, and crystals of the late tephra mainly yield short times of less than ten days (Table S1). Modelling the P traverses provide similar or somewhat longer times of about a week to 50 days. More than 50% of the calculated timescales (Fo, Ni and P modelling) are less than five days, and less than 1.4 days for the early tephra (Fig. 4). Considering that dyke propagation initiated about 10 km below the earth’s surface^27,31,32^ we have estimated magma ascent rates between 0.08 and 1.16 m s^−1^ from the early tephra.

### Crystal record of changing intensive variables, magma convection, and transition towards steady magma flow

A striking feature of the olivine crystals from the earliest tephra is their alternating and sharp variation of high and low Fo and Ni concentrations, which are correlated with the alternating P variations. These observations are not common in olivine crystals from monogenetic volcanoes^16,47–49^, but have been reported in eruptions from Kamchatka (Shiveluch volcano^14^) and kimberlites of South Africa (Benfontein^26^). Such sharp and large compositional variations of Fo, Ni, and P imply significant changes of the intensive variables, which when combined with the skeletal shapes of the crystals and the inferred short timescales, indicate that the olivine grew fast in a very dynamic environment, with significant changes in temperature and *f*O_2_, possibly as the magma was moving towards the surface. Classical magma mixing (e.g. new mafic intrusion in a more evolved reservoir^16^) is unlikely responsible for the recorded cyclic zoning, since there are no clusters of olivine core, mantle, or rim compositions that could be indicative of distinct magma batches and reservoirs^14,16,50^. Instead, we interpret the wide range of olivine compositions and their cyclic variations to reflect crystallization and self-mixing of melts inside a conduit with large temperature and *f*O_2_ gradients (Figs. 2 and S11 and S12).

**Figure 5:**
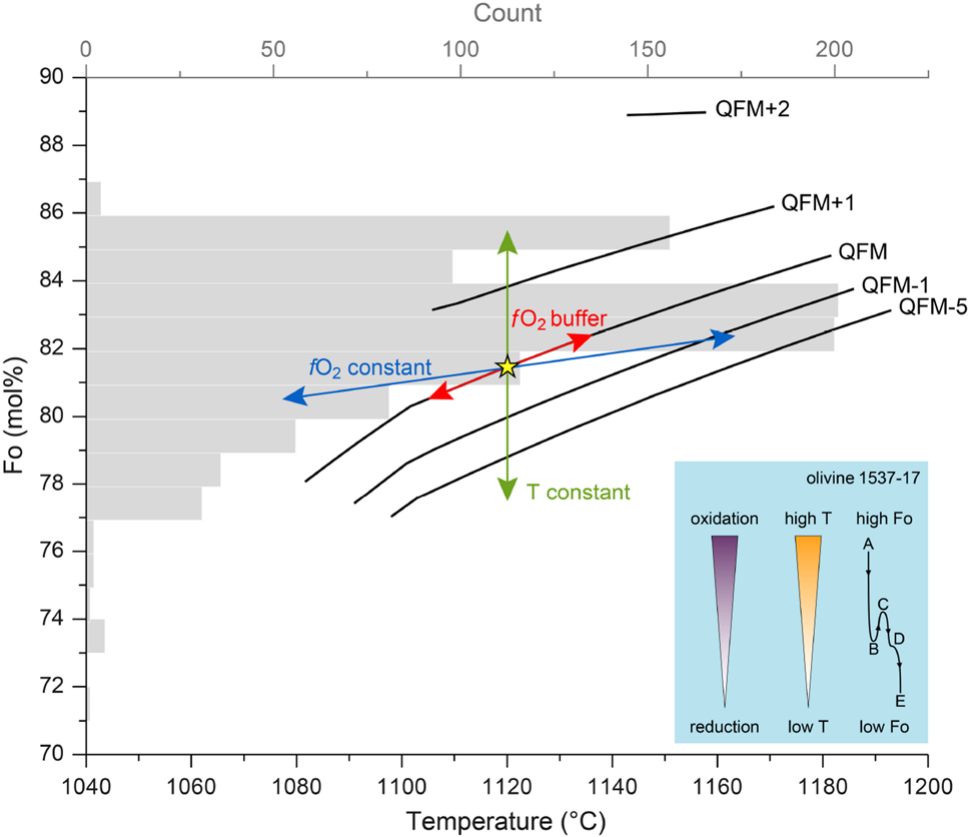
Role of temperature and *f*O_2_ on olivine composition and crystallization trends computed with Rhyolite-MELTS v.1.2.0 (using the most primitive sample, at 3 kbar, 2.5 wt% H_2_O; Table S3). In grey, the olivine Fo histogram from sample 1537 is shown. Yellow star is a hypothetical olivine composition used to discuss the changes in temperature and *f*O_2_ that are required to explain the large and abrupt compositional Fo changes shown by single olivine crystals (e.g. olivine 1537–17 shown in Fig. 3 and represented by letters in inset). We have included three end-member scenarios to explain Fo changes: constant temperature (green arrow), along a *f*O_2_ buffer (red arrow), constant *f*O_2_ (blue arrow). For further details see main text.

The results of Rhyolite-MELTS models (see above) allow us to evaluate the likely ranges in temperature and *f*O_2_ necessary to reproduce the Fo variations in single crystals, which include (Fig. 5): (1) a decrease/increase of ≈ 65 °C at a given *f*O_2_ buffer (e.g. QFM), (2) a decrease/increase of ≈200 °C at a constant *f*O_2_ value (rather than along a buffer), (3) a change in the redox state of the melt of 4 log units (e.g. from QFM + 1 to QFM – 3 or vice versa) at a constant temperature, or (4) a simultaneous change of temperature and *f*O_2_ that did not follow the reaction buffers. Moreover, such changes occurred multiple times, and in both directions, implying more than one cooling and heating (or/and oxidizing and reducing) event. Given the wide range of parameters, and that olivine and spinel grew rapidly and thus not in equilibrium conditions, we cannot establish the exact values of variables that are responsible for the olivine compositional variations. However, our analysis shows that large and rapid changes in temperature and *f*O_2_ and a combination of cooling/heating and oxidation/reduction are needed to explain the zoning of the olivine crystals (and spinel, see below).

We have also attempted to reproduce the range of spinel compositions with the software SPINMELT-2.0^51^, using the same composition as for the Rhyolite-MELTS calculations, and we have explored a range of pressure, water content, and *f*O_2_ (Table S4 and Fig. S5). We found that we were not able to reproduce the spinel compositions within the wide range of investigated variables: natural spinel contains less Mg and Al, and much more Cr and Fe^3+^ than the calculated ones. Notwithstanding this discrepancy, the core-to-rim changes in spinel composition suggest an oxidizing trend involving at least 3 log units of oxygen fugacity (from QFM − 1 to QFM + 2; Fig. S5). The spinel inclusions in the cores and rims of the olivine also record an oxidizing trend (see spinel 16, 17, and 18 hosted in different zoning areas of olivine 14; Fig. S5). This trend has also been found for spinel hosted in fast growing olivine from kimberlites^26^. It seems very likely that there is a relationship between the crystallization of olivine and that of spinel: olivine removes Mg and Fe^2+^ from the melt, generating a local boundary layer enriched in Fe^3+^ and Cr. Variations in the melt composition will affect the liquidus temperature and will be associated with significant changes in the spinel compositions^51^. The spinel may crystallize from this melt with higher Cr and Fe^3+^ and lower Mg and Al, and reduce the *f*O_2_, allowing lower Fo to crystallize in turn. This could explain why the Cr content in the spinel is too high to be in equilibrium with the melt, and at the same time the large variation of Fo and presence of spinel as inclusions in olivine.

**Figure 6:**
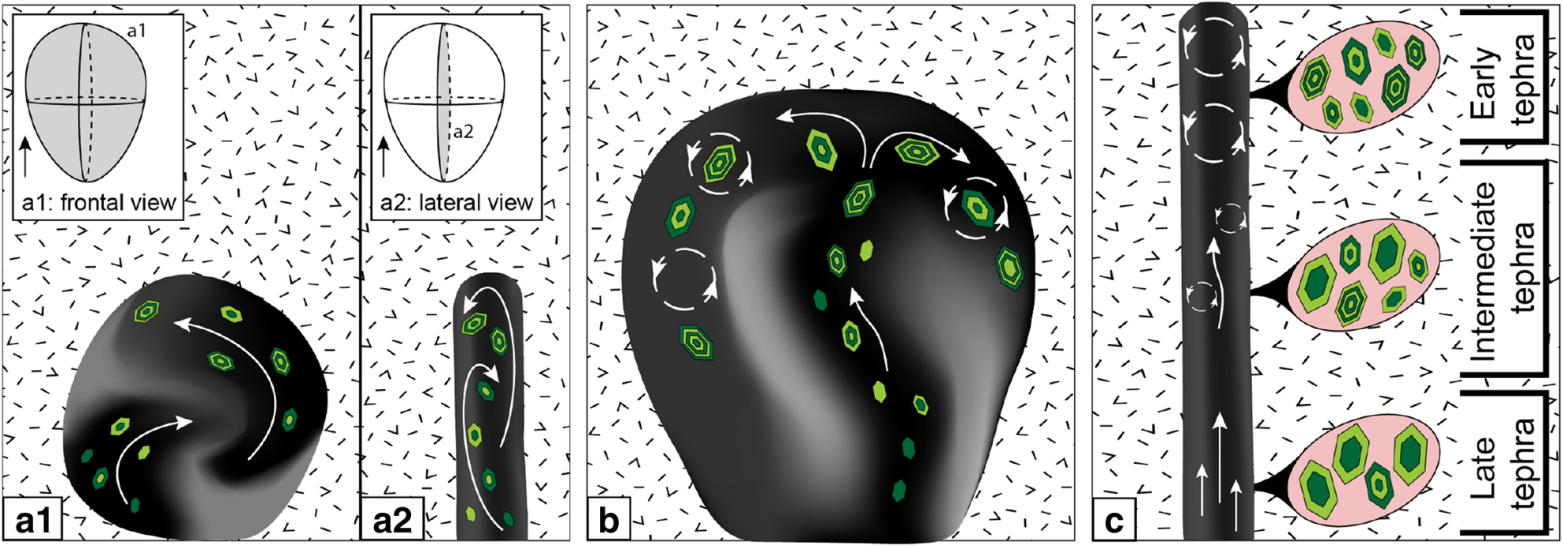
Schematic illustration of the magma flow at different stages of a dyke propagation combined with crystal zoning patterns. (**a**) Dyke inception and (**b**) dyke growth towards the surface. Dyke propagation initiated about 10 km at depth and took < 2 days to reach the surface (ascent rates ≥ 0.1 m s^−1^). During these stages we infer a very dynamic environment with fast olivine growth due to large gradients in temperature and *f*O_2_ that led to magma convection. The result is a collection of complexly zoned crystals (with cyclic Fo, Ni, P zoning in many cases). (**c**) When the dyke is well established, the environment becomes less dynamic and magma flow is likely laminar with crystals growing with a more homogeneous core of lower Fo (late tephra). Olivine crystals from the intermediate tephra record the transition between the two regimes. Model based on petrological data combined with analogue experiments^8^. (**a1**, **b**) Frontal view of the dyke. (**a2**,** c**) Lateral view of the dyke.

We interpret the progressive changes of olivine compositions and zoning patterns (from complex to normally zoned) with the eruption sequence to reflect the transition from an early convective magma transport with large gradients in temperature and *f*O_2_ at the tip and margins of the dyke, towards a more steady and stable laminar magma flow in the later parts of the eruption (Fig. 6). The first erupted crystals (complexly zoned) grew in a convective environment with self-mixing of melt and crystals, without variations in the bulk magma composition (Fig. 6). The magma in contact with the wall-rock cools and becomes more reduced, and heats up and oxidizes when brought back to the centre of the dyke. The thermal gradients at the contact between the intruding dyke and the host rock favours the crystallization of skeletal olivine crystals with complex zoning patterns^23,24^. Fast olivine crystallization leads to the formation of local melt boundary layers, oxidation of the melt, and growth of the spinel. After some time during the first phase of eruption, the conditions of magma transport progressively become more stable, and when the dyke is completely open, the flux of magma changes from turbulent/convective to laminar, and olivine displays normal zoning due to crystal fractionation during ascent to the surface (Fig. 6c). This interpretation is consistent with the calculated timescales of hours to few days, where the complex zoning in olivine is generated by processes occurring in the dyke, and not inside a magma reservoir. The processes occurring in an opening dyke and the transitions from initiation to steady transport appear to be too complex to be investigated numerically. However, such evolution is supported by fluid dynamic experimental models^8^ and petrological data^14,22,26^.

Our observations show an evolution of the crystal population (texture and zoning patterns) along with the eruptive sequence, from more unstable magma transport in the early tephra, to more stable magma flow in the late tephra. Moreover, crystals in the late tephra are very similar to those of the lavas that followed. Therefore, the differences in crystal compositional and textural features, and thus in magma dynamics, preceded the changes in the explosive to effusive eruption dynamics. Thus, detailed tracking of changes in mineral characteristics during eruption sequences could be used to anticipate changes in the eruption style in future eruptions. Additional studies of well-characterized stratigraphic sections of eruption sequences from monogenetic volcanoes^52–54^ combined with detailed crystal zoning observations should allow to test the model proposed here for anticipating changes in eruptive style and help to better understand magma transport towards the surface.

## Methodology

### Samples

Our samples were collected in two trenches (PAR-1536 and PAR-1209) and on a hill (PAR-1537) located < 1.5 km to the southwest of the main cone, chosen to capture the early explosive activity of the eruption. In two of the sections (PAR-1537 and PAR-1536) the contact with the underlying soil was found, providing the complete section of the earliest tephra fall in proximal areas. Samples PAR-1536 are equivalent to samples from site C of Pioli et al. (2008)^5^. The stratigraphic section of tephra immediately above the paleosol corresponds to the first months of the 1943 eruption. These deposits contain the most primitive magma compositions (52.9–55.2 wt% SiO_2_; 5.6–7.5 wt% MgO)^28^. In total we have selected nine units: (PAR-) 1537, 1536A, 1536B, 1536C, 1536D, 1536E, 1209A, 1209B, and 1209C (from bottom to top after stratigraphic and chemical correlation between sites). We have considered a subset of four representative units and divided them into early (1537), intermediate (1536B and 1536E), and late (1209C) tephra samples.

### Analytical methods

We characterized the petrology of the nine tephra units with the scanning electron microscope (SEM) and identified changes throughout the eruptive sequence. Here we report more detailed results of four representative samples (1537, 1536B, 1536E, and 1209C) that have been also analysed with an electron probe micro-analyzer (EPMA).

A polarizing optical microscope, and the JEOL JSM-7800F field emission scanning electron microscope at the Earth Observatory of Singapore were used for petrographic analyses. Backscattered electron (BSE) images, quantitative analyses of the minerals and glass, and five X-ray elemental distribution maps of olivine were obtained using a JEOL JXA 8530F field emission electron probe micro-analyzer (EPMA) at Nanyang Technological University (Singapore). Mineral standards were used for calibration and operating conditions were 15 kV accelerating voltage, 15 to 25 nA beam current, focused beam diameter of about 1 µm, 20–30 s counting time on peak position, and 5 s on each of two background positions. Rim-to-core traverses were performed on olivine, orthopyroxene, and feldspar macrocrysts with a spacing of 1–5 µm between analyses. Individual analyses were performed on microcrysts. Electron backscattered diffraction (EBSD) patterns of olivine to determine the orientation of its crystallographic axes were obtained at the Nanyang Technological University using a JEOL JSM-7800F field emission scanning electron microscope (SEM) equipped with an Oxford NordlysNano detector and Oxford’s AZtec microanalysis software. The modal abundance^55^ of each mineral and the vesicles were estimated from backscattered electron images (BSE) acquired with low magnification (Fig. S1).

### Analysed crystals

In total we analysed 42 olivine (≈ 1,800 analyses, including 17 rim-to-core profiles), six orthopyroxene (≈ 270 analyses, three rim-to-core profiles), three clinopyroxene (≈ 150 analyses, two rim-to-core profiles), 14 plagioclase (≈ 280 analyses including 11 profiles), and 28 spinel (≈ 41 analyses) crystals. Data can be found in Tables S5–S8.

### Diffusion modelling

We modelled Fe–Mg^38^ and Ni^37^ concentration gradients using DIPRA^42^. We applied the anisotropy correction for each crystal and reported the uncertainty on diffusion times that accounts for analytical and temperature uncertainty as calculated by DIPRA. The temperature, pressure, and *f*O_2_ used for modelling are 1,120 ± 30 °C, 3 kbar and QFM + 1. The analytical errors are ± 0.3 for the Fo (mol%) and ± 0.02 for the NiO (wt%). In addition, DIPRA calculates the goodness of the fit (discrepancy). Ni discrepancies are higher because the uncertainty/dispersion in the analytical data is larger. Discrepancies would be reduced between 4 and 5 times by changing the uncertainty from 0.02 to 0.03 wt%. In that case the negative and positive error will increase. We have also modelled two P compositional profiles^39^.

## Supplementary information

Supplementary Information

Supplementary Tables S2–S8

## Data Availability

The authors declare that the data supporting the findings of this study are available within the paper and its supplementary files.
