# Supplementary material for: Crystals reveal magma convection and melt transport in dyke-fed eruptions
Source: Sci Rep. 2020 Jul 15;10:11632. doi: 10.1038/s41598-020-68421-4 (PMC7363826; doi:10.1038/s41598-020-68421-4)
Supplement: Supplementary file 1 — Supplementary Information [file 41598_2020_68421_MOESM1_ESM.pdf]

Crystals reveal magma convection and melt transport in dyke-fed eruptions

Helena Albert[1,2], Patricia Larrea[3,4], Fidel Costa[1], Elisabeth Widom[4], and Claus Siebe[5]

[1]*Earth Observatory of Singapore, Nanyang Technological University, 639798, Singapore*

[2]*Central Geophysical Observatory, Spanish Geographic Institute (IGN), 28014, Madrid, Spain*

[3]*Department of Geology and Andean Geothermal Center of Excellence (CEGA), Facultad de Ciencias Físicas y Matemáticas, Universidad de Chile, Plaza Ercilla 803, Santiago, Chile*

[4]*Department of Geology & Environmental Earth Science, Miami University, Oxford, OH, USA*

[5]*Dpto. de Vulcanología, Instituto de Geofísica, Universidad Nacional Autónoma de México, Mexico City, Mexico*

Supplementary Figures

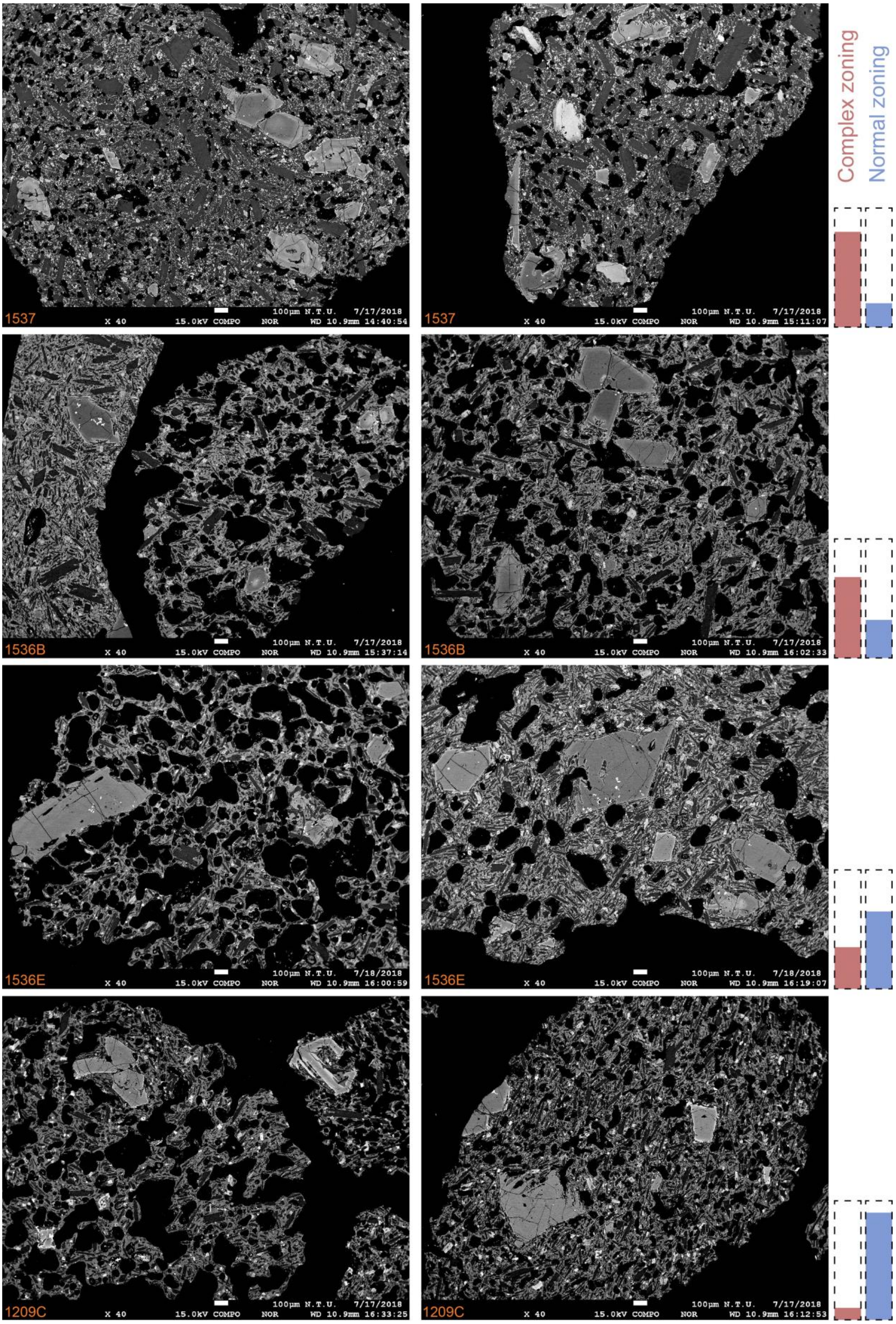

Fig. S1. Zoning type (complex or normal) abundance was estimated from backscattered electron images (BSE) acquired with low magnification. In this figure we show two representative areas for each sample, but in total we used six images for sample 1537, six for sample 1536B, nine for 1536E and nine for 1209C. We used more images for the latter tephra samples since the abundance of crystals decreases. In total we considered 75 crystals for 1537, 33 for 1536B, 31 for 1536E and 41 for 1209.

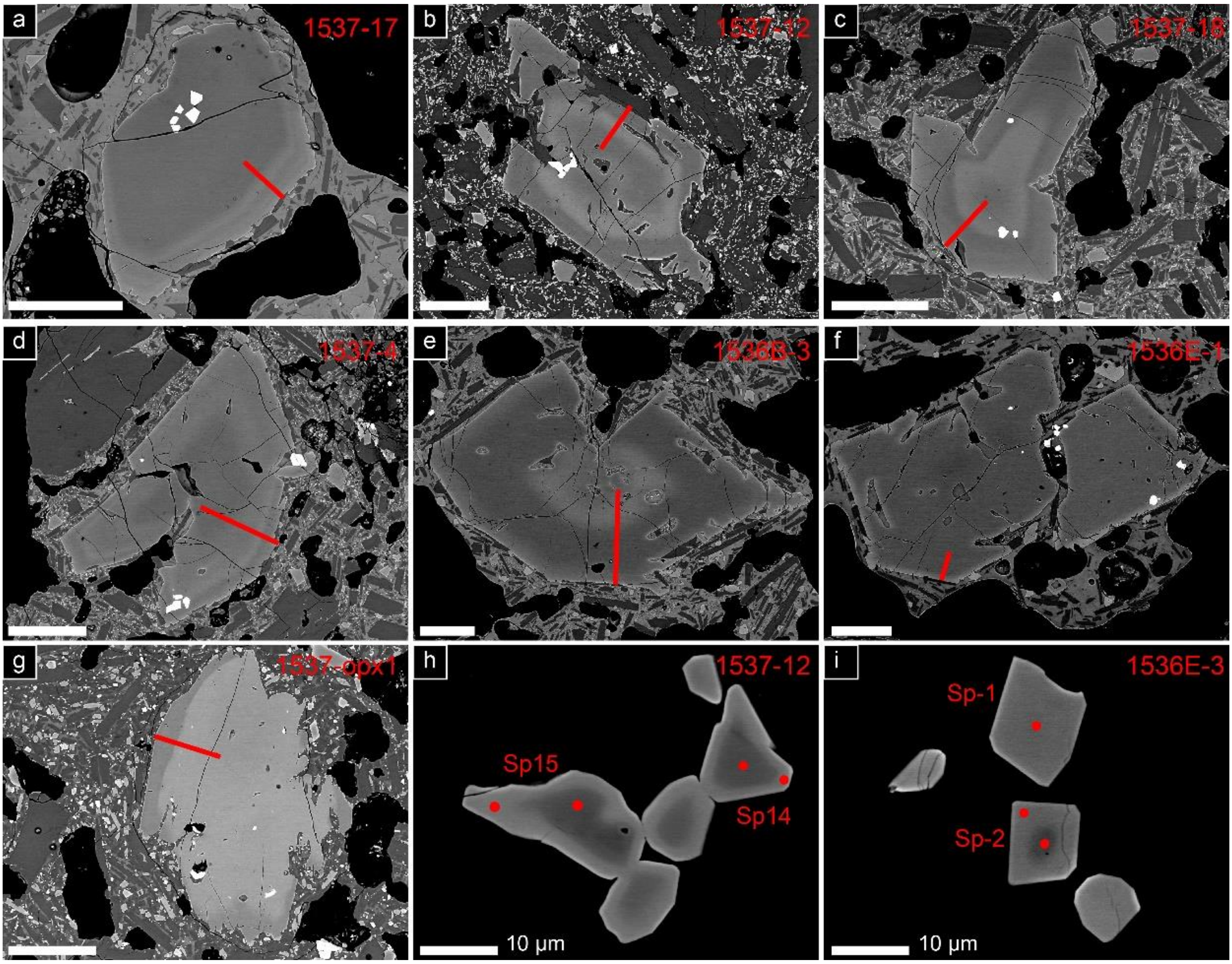

Fig. S2. BSE images of representative **a-f)** olivine, **g)** orthopyroxene and **h-i)** spinel crystals. Compositional traverses and single points are marked in red. The scale is 100 µm, unless specified.

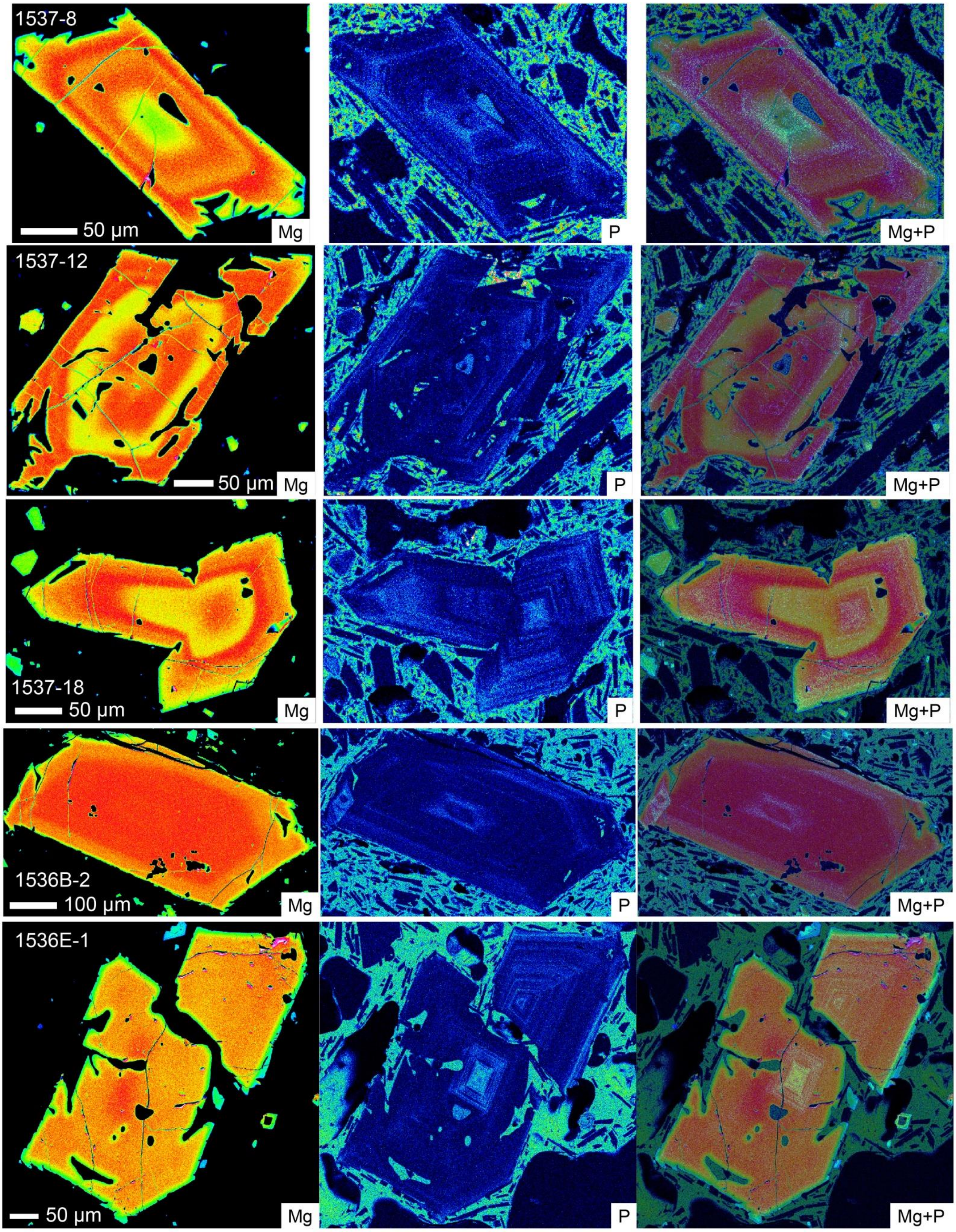

Fig. S3. Magnesium (Mg) and phosphorous (P) X-Ray compositional maps of olivine crystals from the early (samples 1537) and intermediate (1536) erupted tephra. The cyclic Mg and P zoning patterns overlap suggesting that they were generated during growth and not because of magma mixing events. Crystals have different core compositions and different numbers of zoning bands.

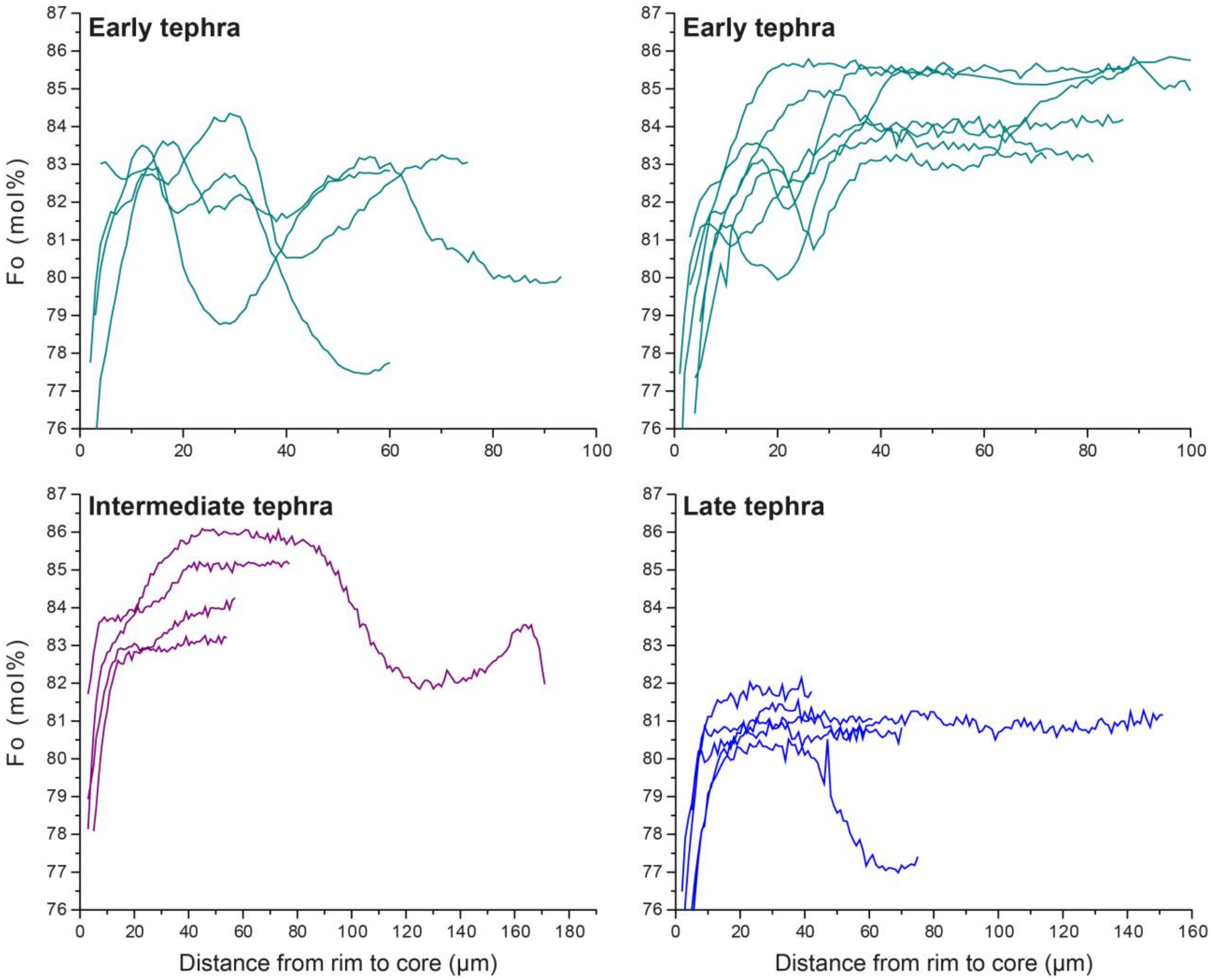

Fig. S4. Olivine compositional profiles of all the analysed crystals from the early, intermediate and late tephra samples (1537, 1536 and 1209C, respectively). Data can be found in Table S5.

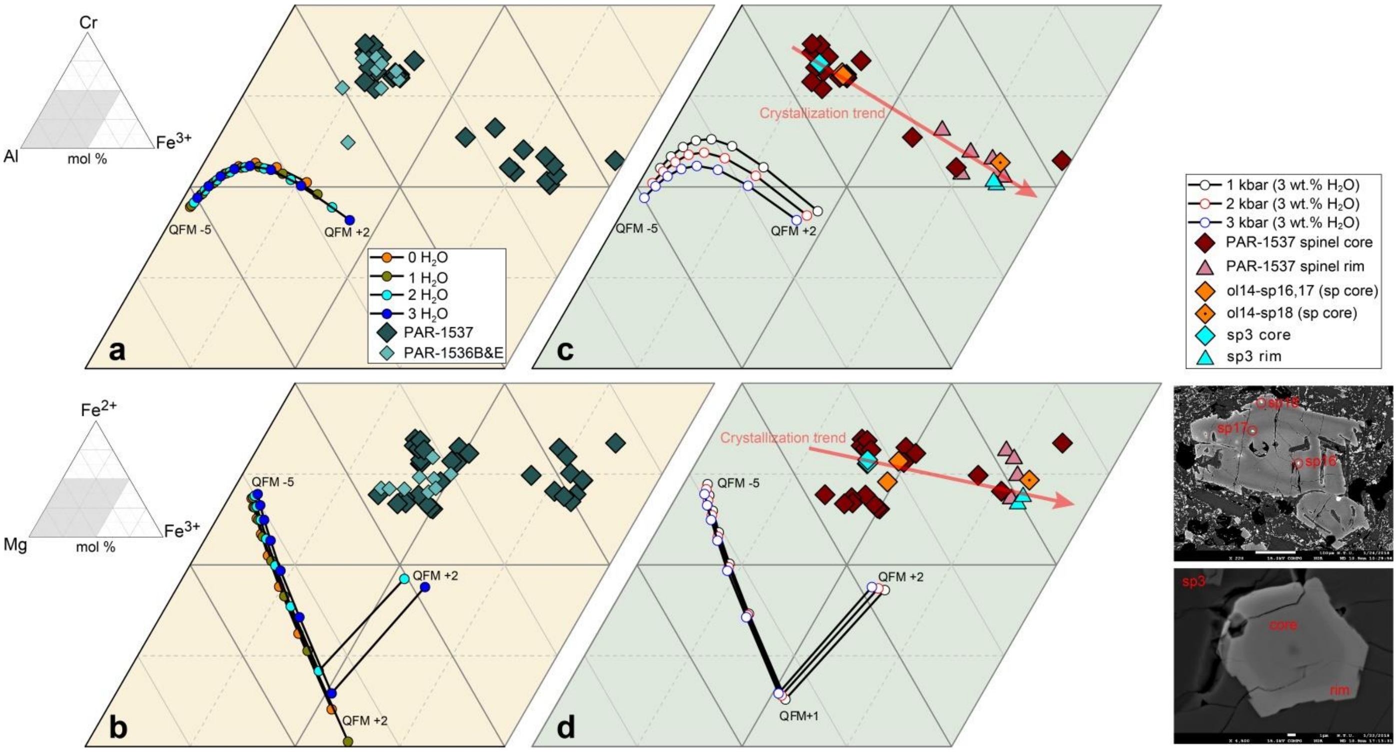

Fig. S5. Olivine hosted spinel compositional diagrams and models according to SPINMELT-2.0 software[1]. **a,b)** Left panels include spinel crystals from early and intermediate samples (1537 and 1536 respectively) for comparison. On the left panels a variation of the H_2O content in the melt has been considered for modelling (P = 3 kbar). As can be observed by the overlapping of the curves, the H_2O content has little influence on the spinel composition. **c-d)** Constant H_2O = 2.5 wt.% has been considered at varying pressure between 1 and 3 kbar. We have highlighted spinels 1537-16, 17 and 18 located at the core, inner and outer rim of olivine 1537-14 (each spinel is in a different Fo band), and spinel 1537-3 which has been analysed at the core and the rim. Most of spinel cores have less Fe^{3+} than the rims. The four spinel cores matching the rim compositions are located in the olivine rims. There is an oxidizing trend recorded by the spinel grains from core to rim (e.g. spinel 3). This trend can also be observed in spinel crystals located in different zoning bands inside the olivine (core, inner and outer rim; e.g. spinel 16, 17 and 18). BSE images of olivine 1537-14 and its hosted spinel crystals (16, 17 and 18 from olivine core to rim) and spinel 1537-3. Spinel 1537-3 is compositionally zoned and shows a concentric pattern that follows the crystal faces and that preserves the external angles (see also Fig. S2). Data can be found in Tables S4 and S7.

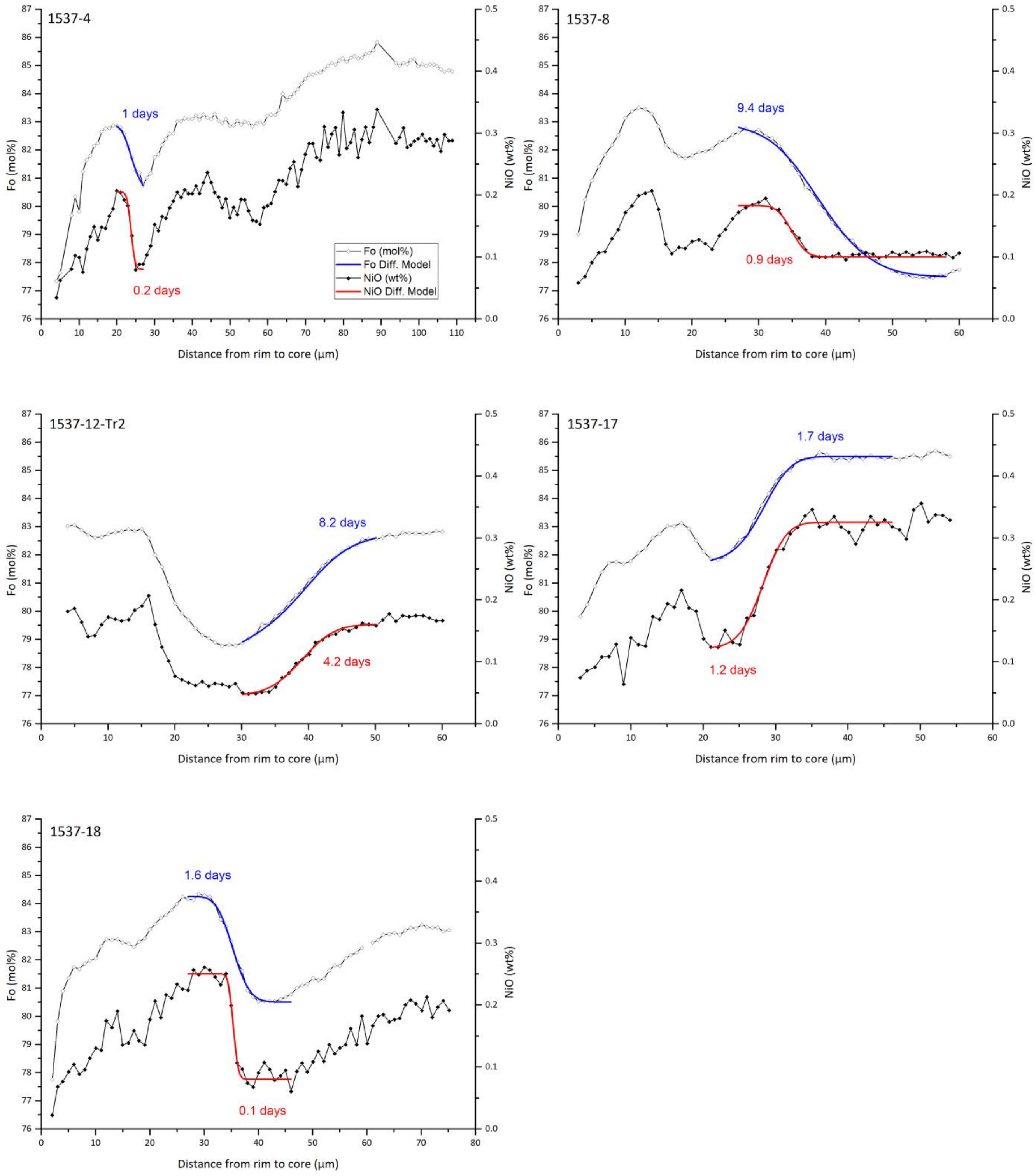

Fig. S6. Diffusion modelling in crystals from the early tephra (sample 1537). Zoning profiles are characterised by cyclic variations in Fo and Ni. We have selected for modelling sectors where compositional plateaus can be identified as initial conditions. Modelling of crystal 1537-8 shows a discrepancy between the Fo and Ni inflection points indicating fast growth.

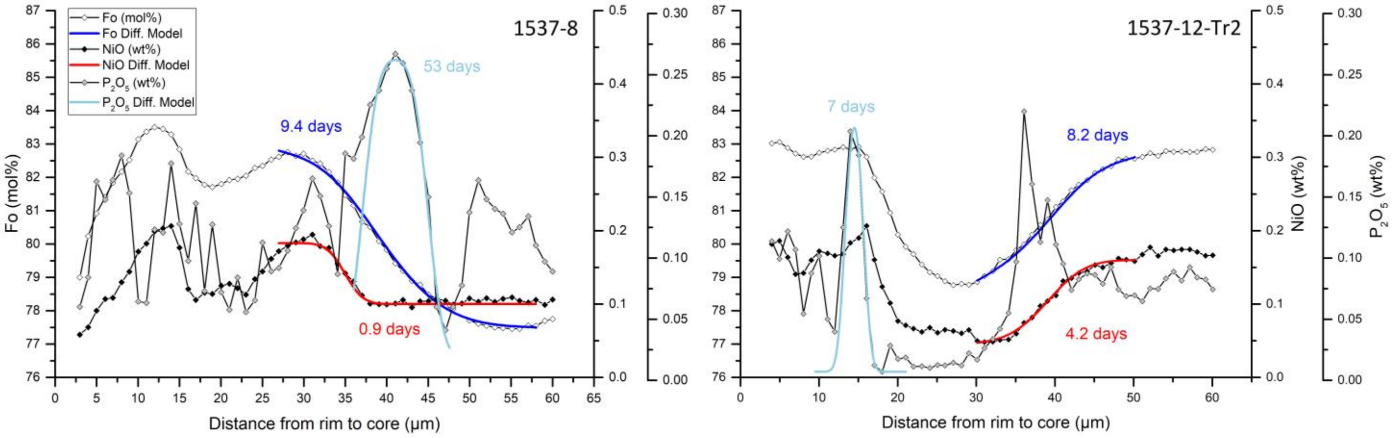

Fig. S7. Diffusion modelling of Fo, Ni and P in two crystals from the early tephra (sample 1537).

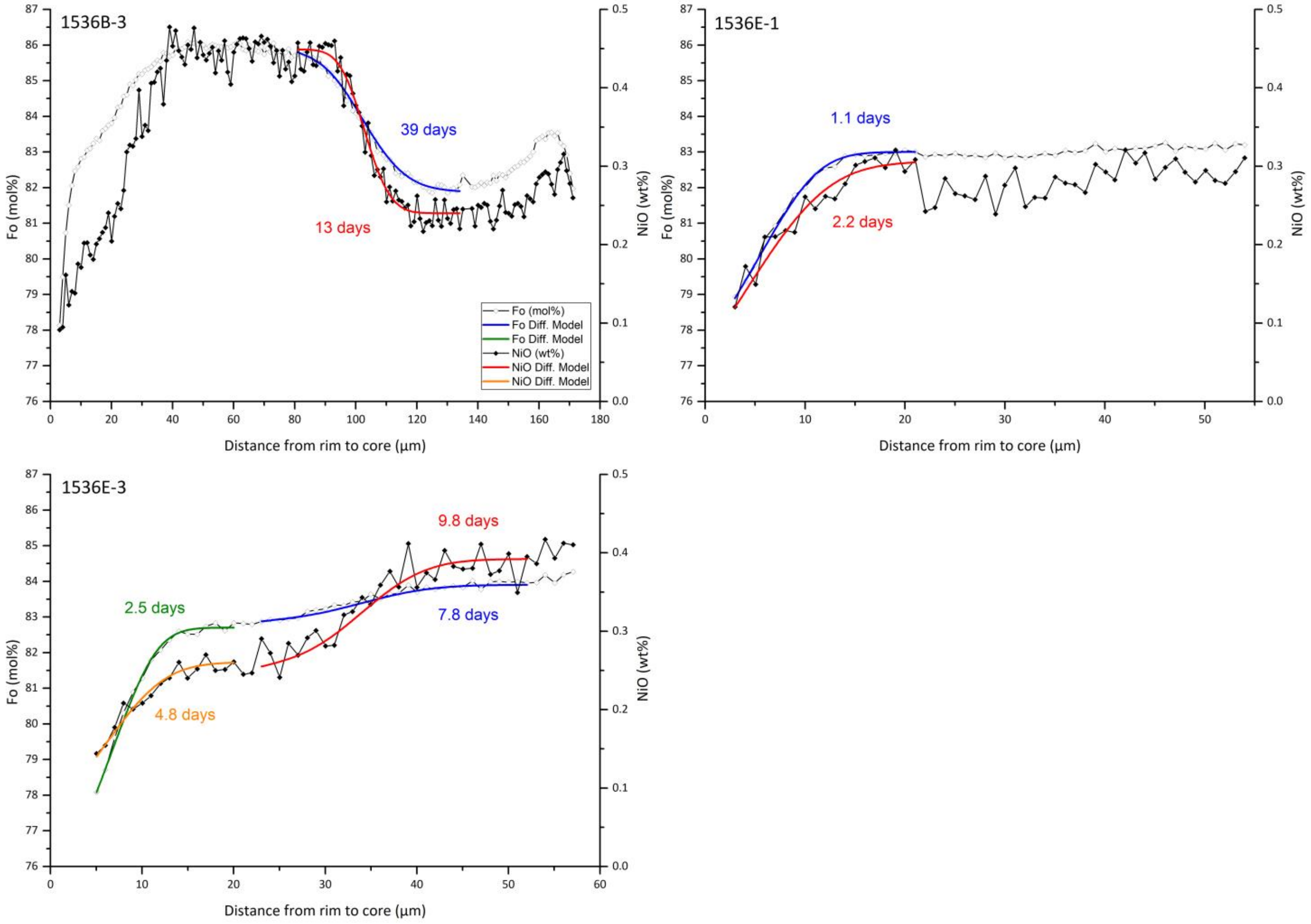

Fig. S8. Diffusion modelling in olivine crystals from the intermediate tephra (sample 1536). We found both complex and normally zoned crystals, reflecting the transition in the flux dynamics inside the dyke.

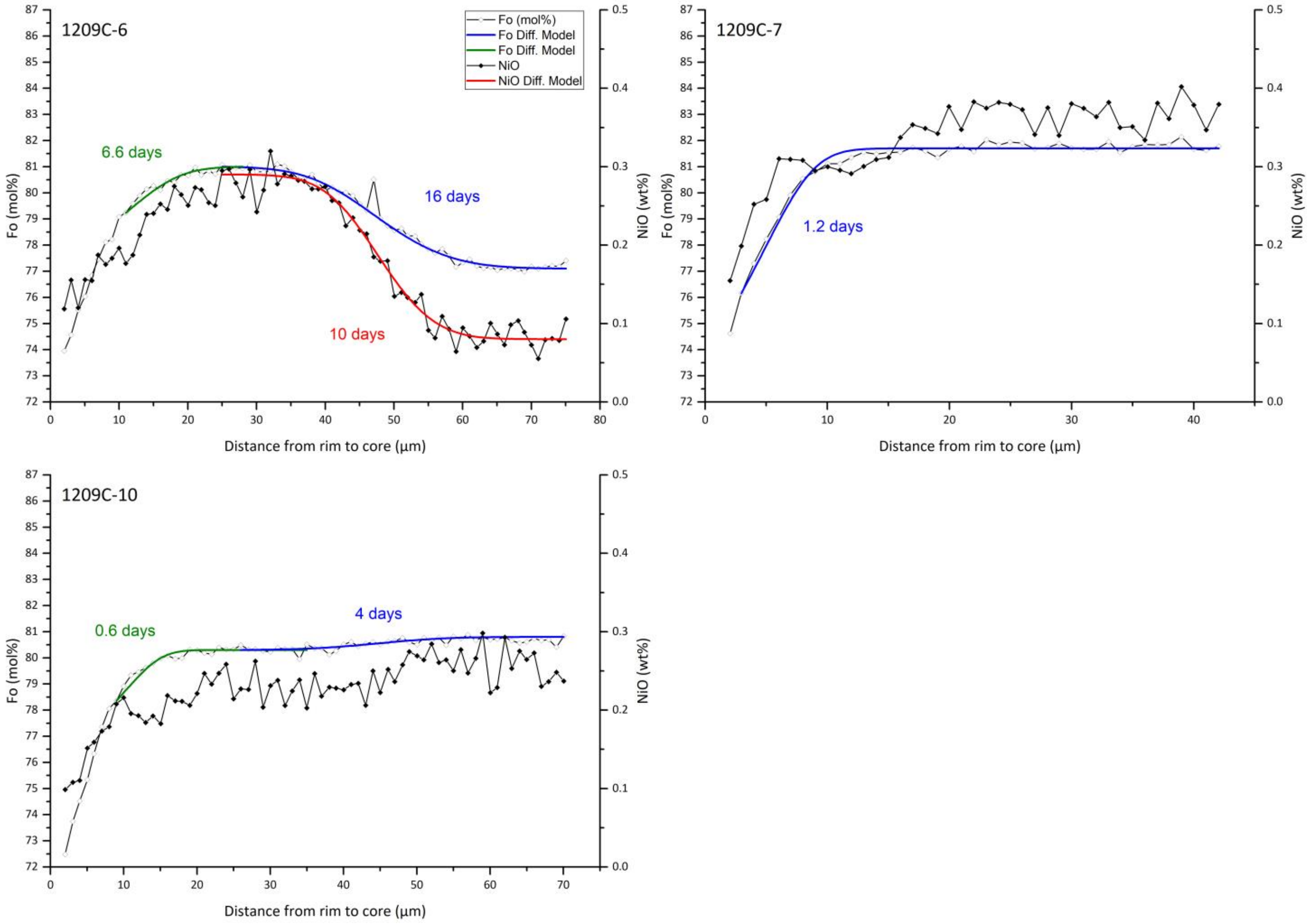

Fig. S9. Diffusion modelling of the late tephra (sample 1209C). Normal zoning at the rim that can be modelled for Fo, but displays a strong discrepancy for the Ni. Olivine crystals are still skeletal indicating rapid growth. The main difference with the previous olivine crystals is that the compositional profiles are not indicating a dynamic environment (small Fo variation and generally normal zoning). However, Ni is not always mirroring the Fo profile, possibly indicating fast growth[2]. We found scarce crystals with a more complex profile, indicating the transition from a convective to laminar flow in the conduit.

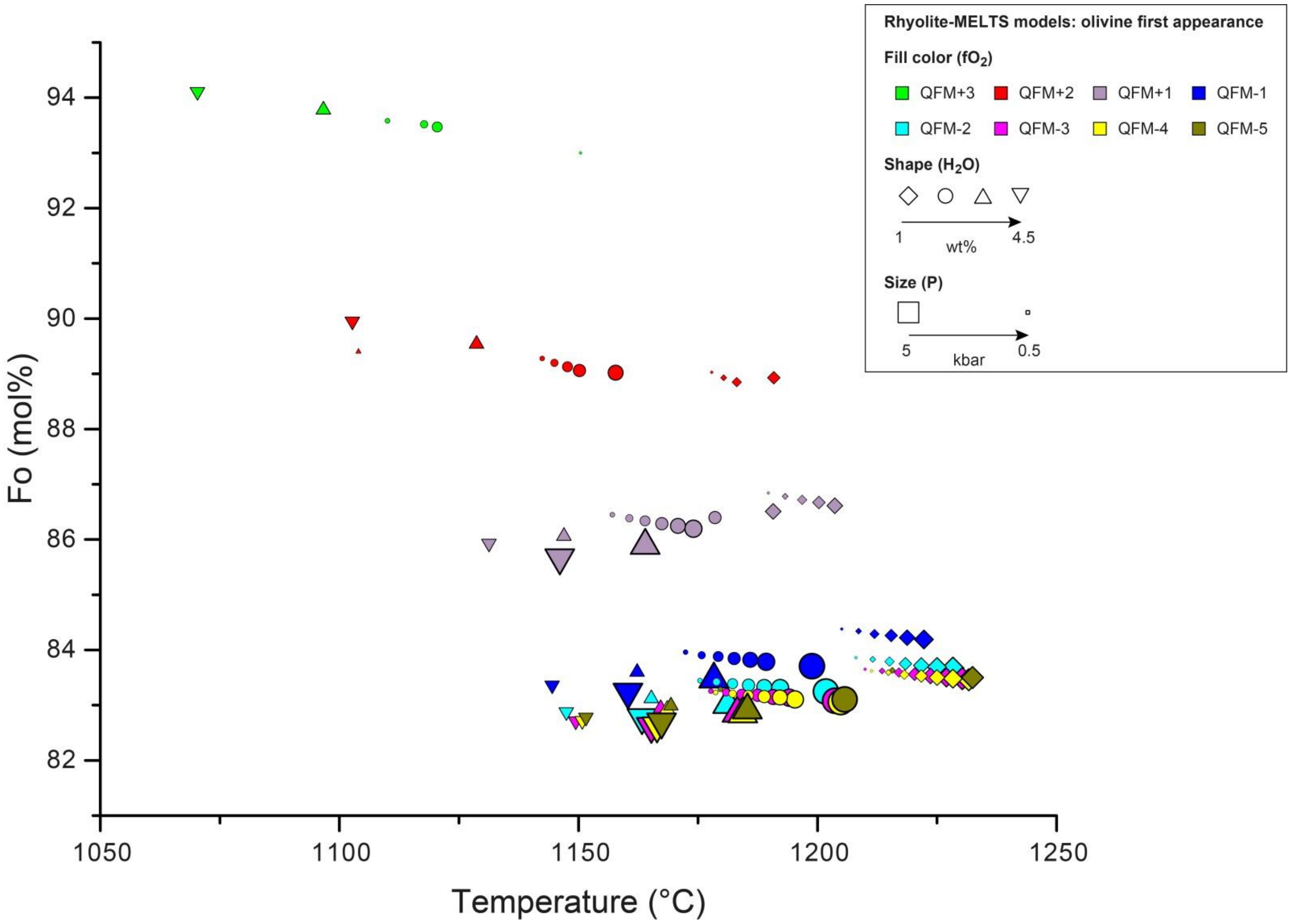

Fig. S10. Isobaric fractional crystallization models calculated with Rhyolite-MELTS v.1.2.0[3,4] at different initial pressures (0.5-5 kbar), with cooling steps of 5 °C, variable water content (1-4.5 wt.%) and different fixed oxygen fugacity buffers ranging from QFM+3 (quartz-fayalite-magnetite) to QFM-5 (Table S2). Each point accounts for the first olivine crystallized according to the models. Changes in the water content and the pressure slightly vary the Fo content, whilst the fO_2 has a strong control on the olivine composition.

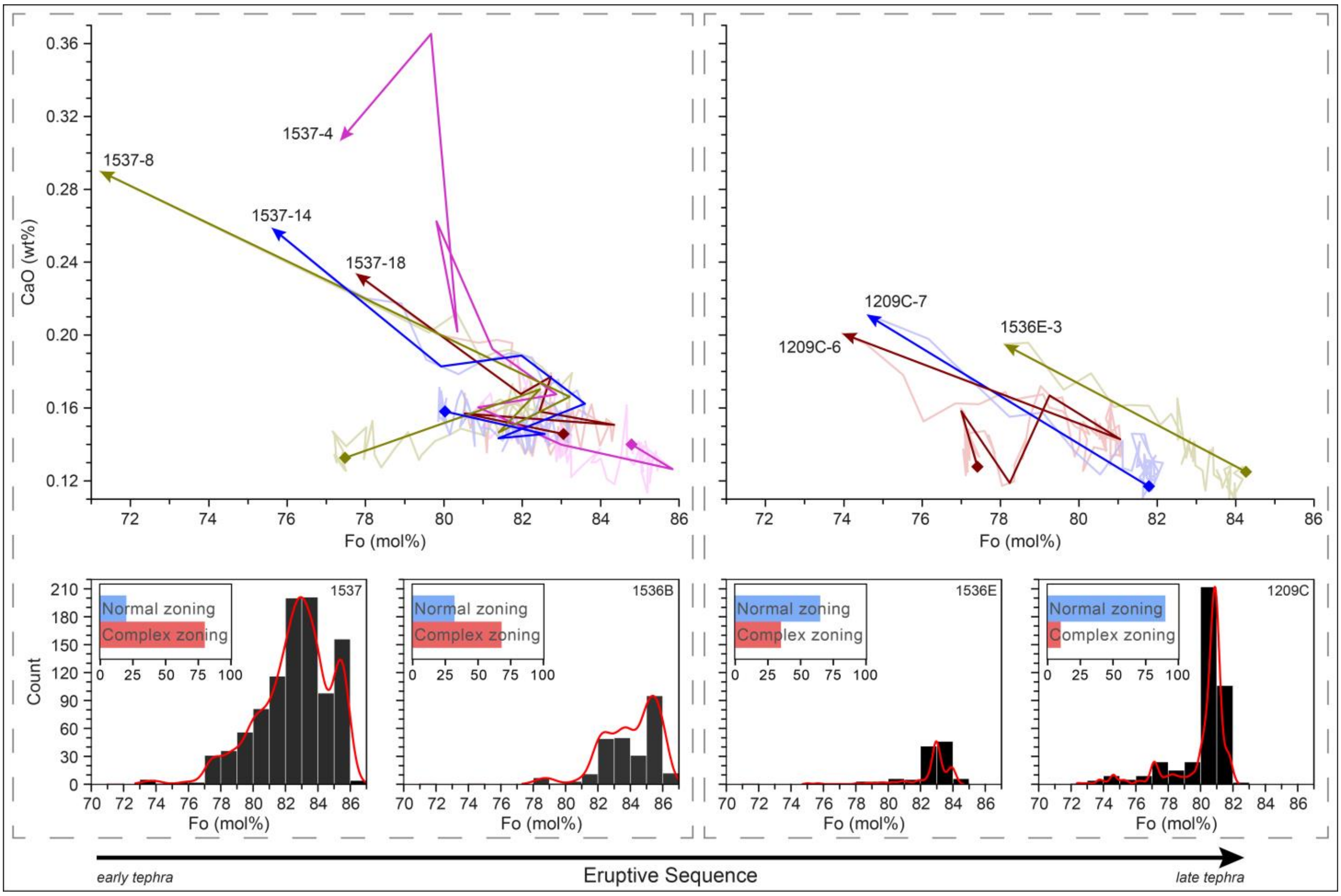

Fig. S11. Fo-Ca variations along compositional profiles of olivine crystals from the early to the late erupted tephra along the eruptive sequence. Measured compositional profiles are overwritten with simplified trends, allowing to follow the tangled trajectories. Crystal cores are represented by a diamond and rims by an arrow. The profiles from sample 1537 are complex, do not correspond to crystal fractionation trends and there is no cluster between the crystal cores or rims. Instead, the latter tephra is characterized by normally zoned crystals with cores at Fo≈82, except for some rare crystal (e.g. 1209C-olivine 6) and rims tend to a common composition.

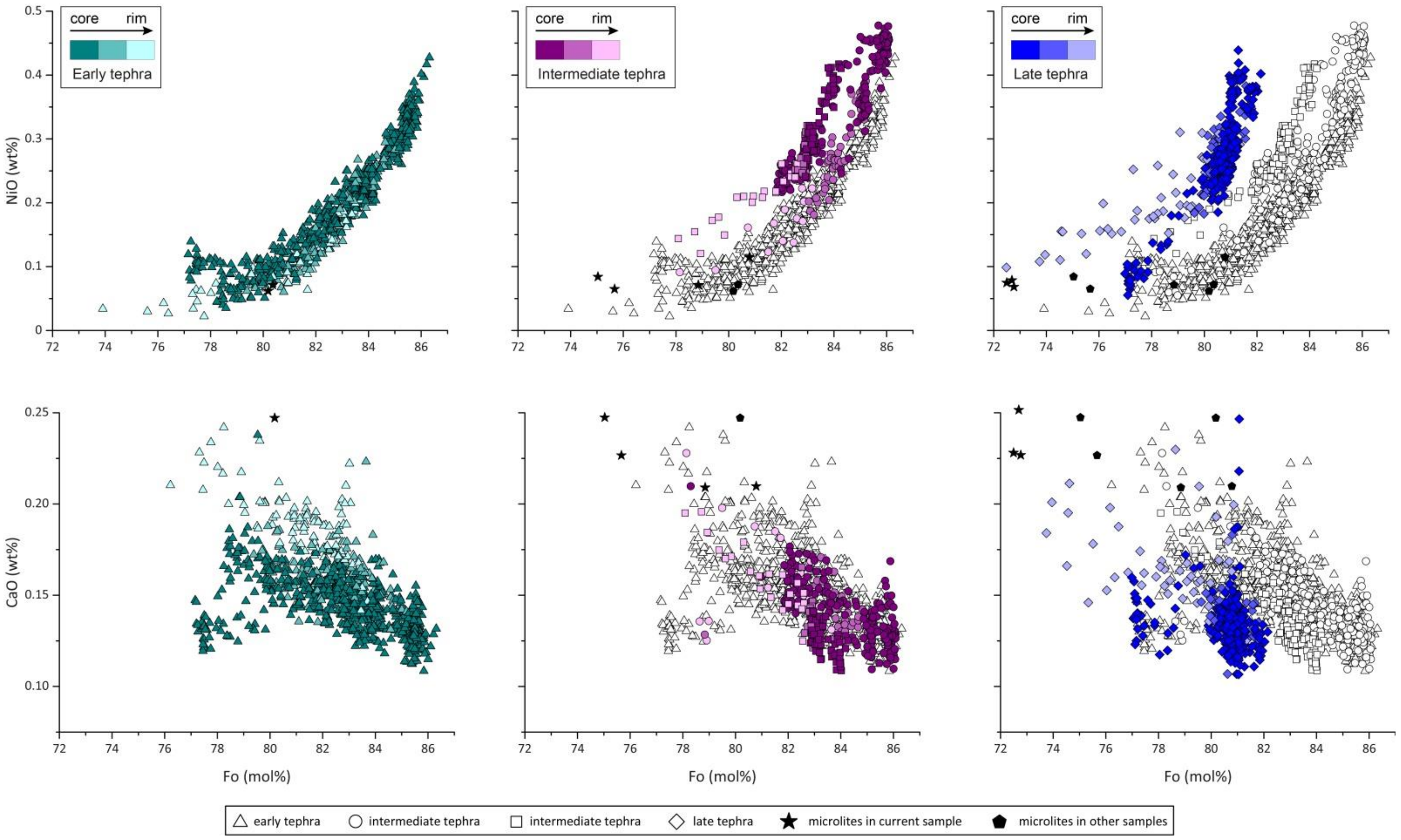

Fig. S12. NiO and CaO wt.% versus Fo [Fo = 100 x Mg/(Mg + Fe) mol%] content of the analysed olivine crystals. Colour intensities vary with distance from core. Early tephra displays a wider core composition than intermediate and late tephra. Data can be found in Table S5.

Table S1. Olivine calculated timescales

Sample	oliv	Profile part	Element	Time (days)	error (-)	error (+)	discr %	Plateau 1	Plateau 2	T (°C)
1537	4	Mantle	Fo	1	0.7	1.2	<1	80.7	82.9	1120±30
1537	4	Mantle	Ni	0.2	0.2	0.3	<1	0.21	0.08	1120±30
1537	8	Core	Fo	9.4	3.7	7.1	<1	77.5	82.8	1120±30
1537	8	Core	Ni*	0.9	0.9	2.4	<1	0.1	0.18	1120±30
1537	8	Core	P	53				0.02	0.264	1120
1537	8	Core	P	35				0.02	0.264	1150
1537	12-Tr2	Core	Fo	8.2	4.9	6	<1	82.8	78.9	1120±30
1537	12-Tr2	Core	Ni	4.2	4.5	8.4	<1	0.05	0.16	1120±30
1537	12-Tr2	Core	P	7				0.009	0.21	1120
1537	12-Tr2	Core	P	4.62				0.009	0.21	1150
1537	17	Core	Fo	1.7	1	1.7	<1	85.5	81.8	1120±30
1537	17	Core	Ni	1.2	0.5	0.7	12	0.32	0.12	1120±30
1537	18	Mantle	Fo	1.6	0.8	1.6	<1	80.5	84.3	1120±30
1537	18	Mantle	Ni	0.1	0.1	0.3	11	0.08	0. 25	1120±30
1536B	3	Core	Fo	39	21	29	2	81.9	85.8	1120±30
1536B	3	Core	Ni	13	5	8	15	0.24	0.45	1120±30
1536E	1	Rim	Fo	1.1	0.7	0.6	<1	83	78.9	1120±30
1536E	1	Rim	Ni	2.2	1.1	1.2	17	0.31	0.12	1120±30
1536E	3	Rim	Fo	2.5	1.5	1.8	<1	82.7	78.1	1120±30
1536E	3	Rim	Ni	4.8	2.2	3.5	7	0.26	0.14	1120±30
1536E	3	Core	Fo	9.8	10.5	18.9	<1	83.9	82.8	1120±30
1536E	3	Core	Ni	7.8	3.4	4.1	24	0.39	0.25	1120±30
1209C	6	Core	Fo	16	6	7	2	77.1	81	1120±30
1209C	6	Core	Ni	10	3	6	18	0.08	0.29	1120±30
1209C	6	Rim	Fo	6.6	4.7	4.5	<1	81	79.2	1120±30
1209C	7	Rim	Fo	1.2	0.5	0.7	10	81.7	76.2	1120±30
1209C	10	Core	Fo	4	3.7	3.7	5	80.8	80.3	1120±30
1209C	10	Rim	Fo	0.6	0.4	0.8	8	80.3	78.3	1120±30

*Fo and Ni inflection points are different.

References

1. Nikolaev, G. S., Ariskin, A. A. & Barmina, G. S. SPINMELT-2.0: Simulation of Spinel–Melt Equilibrium in Basaltic Systems under Pressures up to 15 Kbar: II. Description of the Program Package, the Topology of the Cr-spinel–Melt Model System, and Petrological Implications. *Geochemistry Int.* **56**, 125–135 (2018).

2. Costa, F., Dohmen, R. & Chakraborty, S. Time Scales of Magmatic Processes from Modeling the Zoning Patterns of Crystals. *Rev. Mineral. Geochemistry* **69**, 545–594 (2008).

3. Gualda, G. A. R., Ghiorso, M. S., Lemons, R. V & Carley, T. L. Rhyolite-MELTS : a Modified Calibration of MELTS Optimized for Silica-rich , Fluid-bearing Magmatic Systems. **53**, 875–890 (2012).

4. Ghiorso, M. S. & Gualda, G. A. R. An H2O–CO2 mixed fluid saturation model compatible with rhyolite-MELTS. *Contrib. to Mineral. Petrol.* **169**, 1–30 (2015).

5. Ferracutti, G. R., Gargiulo, M. F., Ganuza, M. L., Bjerg, E. A. & Castro, S. M. Determination of the spinel group end-members based on electron microprobe analyses. *Mineral. Petrol.* **109**, 153–160 (2015).
